# Committee on Ethical Issues - Preliminary results of survey on ethics in psychiatric practice

**DOI:** 10.1192/j.eurpsy.2023.158

**Published:** 2023-07-19

**Authors:** M. Schouler-Ocak

**Affiliations:** Psychiatric University Clinic of Charité at St. Hedwig Hospital, Berlin, Germany

## Abstract

**Abstract:**

The code of ethics of the EPA intends to guide the ethical practice of psychiatry by offering a comprehensive approach to the ethical challenges in the field. It highlights universal ethical principles and considers their application to the specific practice of psychiatry. Ethical questions in psychiatric practice are manifold: tensions between respect for autonomy versus care and protection from harm, problems with coercive therapy and capacity for judgement etc. To receive more information, the Committee on Ethical Issues conducted a survey on “Ethics in psychiatric practice” to collect information from inpatient treatment settings of individual wards in psychiatric hospitals Europe-wide on following topics:

- Experiences and practices addressing ethical conflicts and malpractice from the personal perspective of health care workers in these settings
- Identification and engagement with violence
- Measures for the reduction of restraints and coercion (violation of the autonomy of patients)

In this talk, the preliminary results of this survey will be presented and discussed.

**Disclosure of Interest:**

None Declared

